# Fast Regulation of Hormone Metabolism Contributes to Salt Tolerance in Rice (*Oryza* *sativa* spp. Japonica, L.) by Inducing Specific Morpho-Physiological Responses

**DOI:** 10.3390/plants7030075

**Published:** 2018-09-15

**Authors:** Elide Formentin, Elisabetta Barizza, Piergiorgio Stevanato, Marco Falda, Federica Massa, Danuše Tarkowskà, Ondřej Novák, Fiorella Lo Schiavo

**Affiliations:** 1 Department of Biology, University of Padova, 35131 Padua, Italy; elisabetta.barizza@unipd.it (E.B.); federica.massa@phd.unipd.it (F.M.); fiorella.loschiavo@unipd.it (F.L.S.); 2 Department of Agronomy, Animals, Natural Resources and Environment-DAFNAE, University of Padova, 35020 Legnaro (Padova), Italy; stevanato@unipd.it; 3 Department of Molecular Medicine, University of Padova, Viale G.Colombo 3, 35121 Padova, Italy; marco.falda@unipd.it; 4 Laboratory of Growth Regulators, Centre of the Region Haná for Biotechnological and Agricultural Research, Institute of Experimental Botany AS CR & Faculty of Science, Palacký University, Šlechtitelů 27, CZ-78371 Olomouc, Czech Republic; tarkowska@ueb.cas.cz (D.T.); novako@ueb.cas.cz (O.N.)

**Keywords:** *Oryza sativa*, salt stress, phytohormones, phenotypic plasticity, RNA sequencing

## Abstract

Clear evidence has highlighted a role for hormones in the plant stress response, including salt stress. Interplay and cross-talk among different hormonal pathways are of vital importance in abiotic stress tolerance. A genome-wide transcriptional analysis was performed on leaves and roots of three-day salt treated and untreated plants of two Italian rice varieties, Baldo and Vialone Nano, which differ in salt sensitivity. Genes correlated with hormonal pathways were identified and analyzed. The contents of abscisic acid, indoleacetic acid, cytokinins, and gibberellins were measured in roots, stems, and leaves of seedlings exposed for one and three days to salt stress. From the transcriptomic analysis, a huge number of genes emerged as being involved in hormone regulation in response to salt stress. The expression profile of genes involved in biosynthesis, signaling, response, catabolism, and conjugation of phytohormones was analyzed and integrated with the measurements of hormones in roots, stems, and leaves of seedlings. Significant changes in the hormone levels, along with differences in morphological responses, emerged between the two varieties. These results support the faster regulation of hormones metabolism in the tolerant variety that allows a prompt growth reprogramming and the setting up of an acclimation program, leading to specific morpho-physiological responses and growth recovery.

## 1. Introduction

The world is facing increasingly difficult challenges in crop production due to the rapid growth in population, increased linked food demand, and climate change. For these reasons, there is a need to develop productive crops able to resist a wide range of biotic and abiotic stresses [1]. Soil salinity is a major constraint for crop production worldwide, particularly in agricultural lands close to the sea that are threatened by salt intrusion due to the rise in sea levels [2]. Salinization affects at least 33% of arable land, and more areas are expected to deteriorate in coming years because of global climate change and human activities [3].

Rice is a staple food for more than half the world population, and provides a good model for genetic studies on crop adaptation to environmental stresses [4]. Unfortunately, rice is the most salt-sensitive species among the cereals [5]. About 75% of the rice is produced in paddy fields located near the delta of the main rivers, which are being seriously threatened by the increase in soil salinization. Selecting rice varieties with an improved level of salinity tolerance could significantly contribute to maintaining high levels of productivity.

A vast amount of literature addressing the salt stress response in plant has been published [2]. Recently, some molecular players involved in mechanisms of salt tolerance have been unveiled in several plant species [6,7], including rice [8,9,10]. Despite these efforts, however, few fundamental findings have been successfully applied to crop plants [11].

Hundreds of different *Oryza sativa* ssp. japonica varieties are cultivated worldwide, which were selected for their high productivity. The goal is now to achieve an omni-comprehensive knowledge of salt tolerance in rice and to transfer this knowledge into effectively salt-tolerant and highly-productive plants.

Plants are sessile organisms that evolved a plethora of mechanisms to cope with or avoid unfavorable environmental conditions. Phenotypic plasticity is the biggest ally in shaping the plant body in response to interactions with the environment [12] and in escaping from stresses (shade or submergence avoidance [13,14]) or seeking for nutrients (halotropism [15]).

High soil salinity impairs rice plant growth by reducing the number of tillers, thus affecting productivity. The growth restriction appears at the whole plant level and is due to an initial osmotic response, aimed at the reduction in water loss and subsequently to ion toxicity that affects main metabolic pathways (i.e., photosynthesis). Sensitive plants usually undergo an anticipated senescence and death [5,16].

Plant development is regulated by hormones and it is now accepted that phytohormones play a fundamental role in the interaction with the environment. Clear evidence exists for a role of hormones in the plant stress response, either biotic or abiotic [17]. In addition, increasing amounts of data are supporting that interplay and cross-talks among different hormonal pathways are of vital importance in abiotic stress tolerance [17,18,19,20,21]. Fine tuning of different hormones at the cellular level is important in building effective developmental responses. Plant cells possess different mechanisms to control hormonal levels, i.e., the regulation of their synthesis, transport, and conjugation or degradation as well as the efficiency of signal perception and transduction.

The role of the stress hormone abscisic acid (ABA) has been described widely not only in Arabidopsis but also in crop plants [22,23,24]. ABA is involved in the signaling of water deficit (salinity, drought), perceived at the root level, and in the triggering of water-saving mechanisms, such as stomatal closure [25,26], leaf expansion limitation [27,28], and root architecture modulation [18,29,30,31,32].

Recently, the ABA signaling pathway in response to water stress was reported to be cross-linked with those of other hormones: ethylene, cytokinins, and auxins [18]. The interplay among all these hormones explain the shoot growth impairment observed in salinized tomato plants [33].

Ethylene has been reported to have a dual role by positively or negatively affecting plant survival under abiotic stress. Ethylene receptors have been shown to interplay with ABA production and, as a consequence, delay germination of salinized Arabidopsis seeds or survival increase of submerged rice plants can be induced [34].

Cytokinins (CKs), along with auxin, are involved in the maintenance of both shoot and root meristems by stimulating stem cell division [35]. Contrasting data, however, were reported on the role of CKs in abiotic stress response that support both negative and positive effects [36,37]. Studies on the activity of CKs receptors demonstrated an interplay with ABA in transducing abiotic stress signals [38], which in leaves interferes with ABA-induced stomatal closure [39].

Auxin is mainly involved in development and determining the shape of the plant body by controlling cell division and developmental patterning. The action of auxin is strictly related to its concentration and the direction of growth of an emerging organ is determined by auxin gradients. The maintenance of the cellular optimum concentration is achieved through the regulation of biosynthesis, transport, perception, and signaling of the hormone. The alteration of auxin gradients can indeed improve salt tolerance by stimulating the growth and maintaining the biomass even in stress conditions. Unfortunately, whether Arabidopsis plants overproducing IAA showed drought tolerance [40]—rice plants with lower IAA contents either resulted sensitive or tolerant to salt and drought [41,42].

Gibberellins and brassinosteroids, implicated as well as auxin in cell division, have been reported to play a role in salt tolerance by helping in the recovery phase [43] or by interplaying with reactive oxygen species (ROS) [44] and other hormones [17]. Finally, jasmonic acid and salicylic acid have been reported to cooperate in abiotic stress tolerance by interacting with other hormonal pathways and reducing oxidative stress [45,46]

Hormone level manipulation seems a promising approach for obtaining new varieties with improved salt tolerance. However, incomplete knowledge on the intricate crosstalk between the different hormonal pathways is hampering the efforts. More knowledge is needed to complete our understanding of hormone-driven salt tolerance mechanisms.

In this work, we report changes in hormone metabolism induced in two rice varieties differing in salt sensitivity after exposure to salt stress, in order to understand how variations result in different responses that contribute to initiate an adaptive program in the tolerant variety. The hormonal changes were evaluated initially at the transcriptomic level. By means of a genome-wide transcriptional analysis previously performed on leaves and roots of three-day treated/untreated Vialone Nano (VN) sensitive and Baldo tolerant variety [47], all the genes related to hormone metabolism were gathered in hormone-related functional categories and the differences in the expression profile of genes of these categories are reported for the two varieties. Then, the variation in hormonal contents was measured in leaves, stem, and root at one and three days after salt treatment and correlated with the transcriptomic data. All the results support the faster remodeling of hormone metabolism in the tolerant variety that allows a prompt growth reduction, a condition required for initiating an acclimation program leading to specific morpho-physiological features.

## 2. Results and Discussion

### 2.1. Transcriptional Changes in Hormone-Related Functional Categories

**Figure 1:**
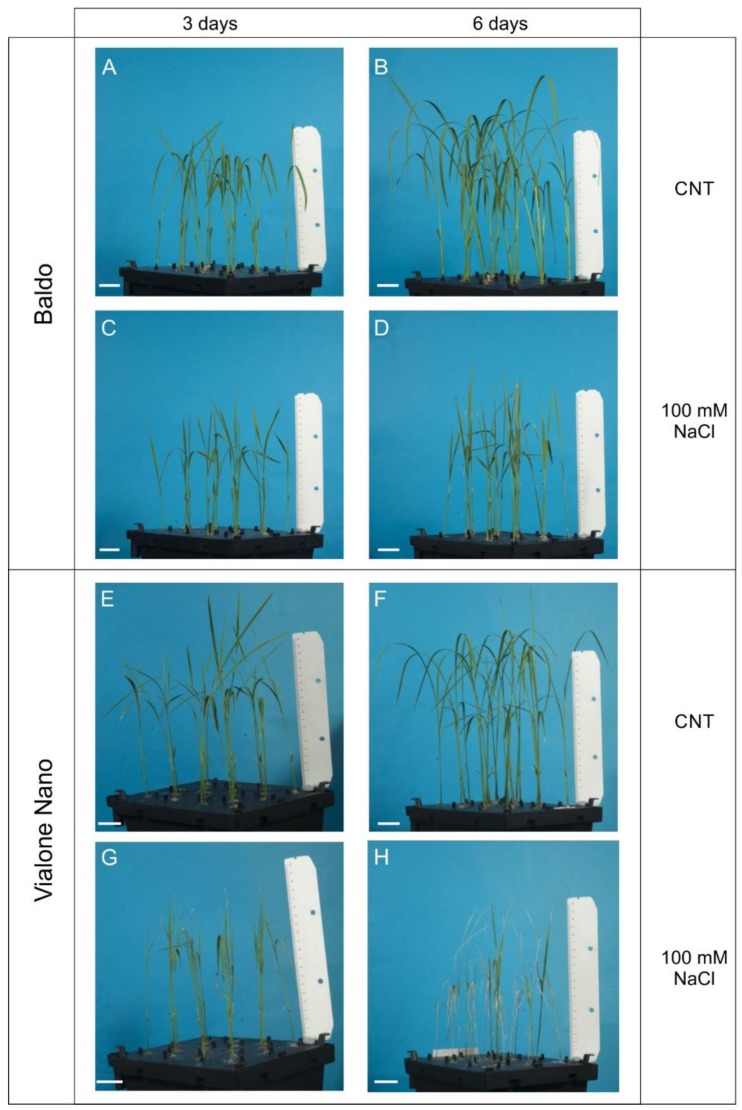
Baldo and Vialone Nano plants grown in hydroponics. (**A,B,E,F**) Untreated plants (CNT). (**C,D,G,H**) Plants at stage V2 of growth were treated with a saline solution containing 100 mM NaCl, for (**C**,**G**) three or (**D**,**H**) six days. Baldo-treated plants showed a resuming of growth at six days (**D**). (**G**) VN plants showed the yellowish of second leaves at day three and (**H**) died after six days of treatment. Bar = 3 cm.

In previous works, we explored the salt tolerance/sensitivity of two Italian rice varieties of great economic relevance for both the internal (Vialone Nano, VN, salt sensitive) and external (Baldo, salt tolerant) Italian markets [47,48]. We demonstrated that prompt H_2_O_2_ signaling at root level was of most importance in inducing salt tolerance in the Baldo variety and that a delay in response led the sensitive variety VN to a chaotic response and leaf senescence (Figure 1).

**Figure 2:**
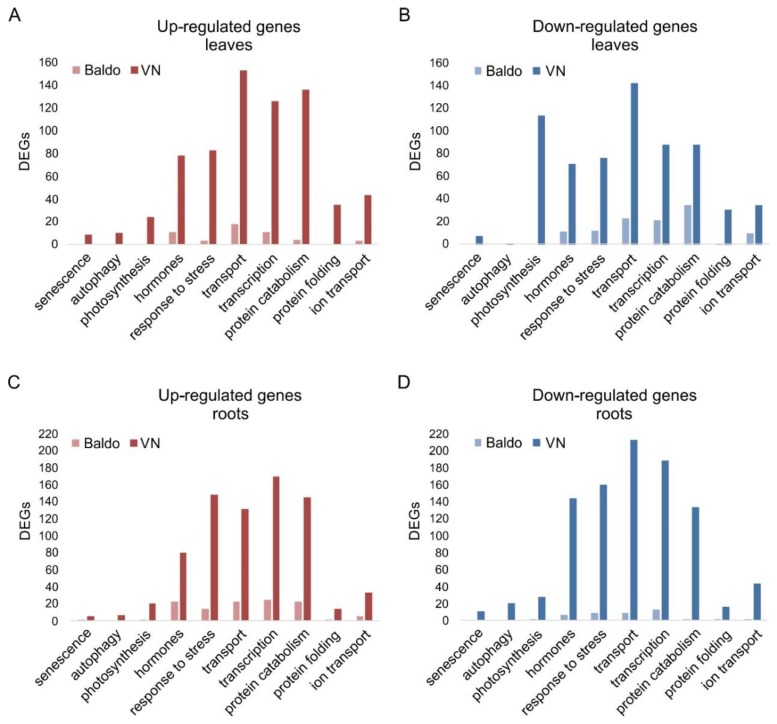
Differentially expressed genes (DEGs) grouped by functional categories. DEGs were re-annotated by using Argot2 tool and grouped in functional categories basing on their biological process ontology terms. (**A**,**B**) Up- and down-regulated genes in leaves of three-day treated plants. (**C**,**D**) Up- and down-regulated genes in roots of three-day treated plants.

Genome-wide transcriptional analyses performed on leaves and roots of three-day treated/untreated VN and Baldo plants indicated that sensitive plants responded with a wider range of responses by regulating the genes regulated by tolerant plants by four-fold (Figure 2). Sensitive plants at this timepoint were undergoing a leaf senescence process, as demonstrated by the modulation of genes related to photosynthesis, autophagy, senescence, and protein catabolism. In particular, most of genes involved in photosynthesis, such as those encoding chlorophylls and photosystems subunits, were down-regulated (Figure 2B), indicating the programed dismantling of photosynthetic apparatus. This result was in line with our previous findings that VN plants (and cultured cells) under salt stress initiated a programmed cell death (PCD) response and leaf senescence [47,48]. On the contrary, tolerant Baldo plants regulated few genes that were grouped into stress response, transport, hormones, transcription, and protein catabolism categories (Figure 2A,C). Notably, photosynthesis seemed unaffected and no one gene was found in the categories related to cell death/senescence, i.e., senescence and autophagy. Genes encoding proteins known to be involved in the positive response to oxidative and osmotic stresses, i.e., peroxidases, drought responsive transcription factors (DREBs), and aquaporins, were down-regulated in Baldo leaves. In roots, several genes for peroxidases and universal stress proteins were instead up-regulated. This suggested that after three days of stress, roots of tolerant plants were still suffering, whereas leaves already overcame the stress.

Interestingly, in the tolerant variety, one of the most represented categories was the one related to hormones, both in leaves (Figure 2A,B) and roots (Figure 2C,D).

**Figure 3:**
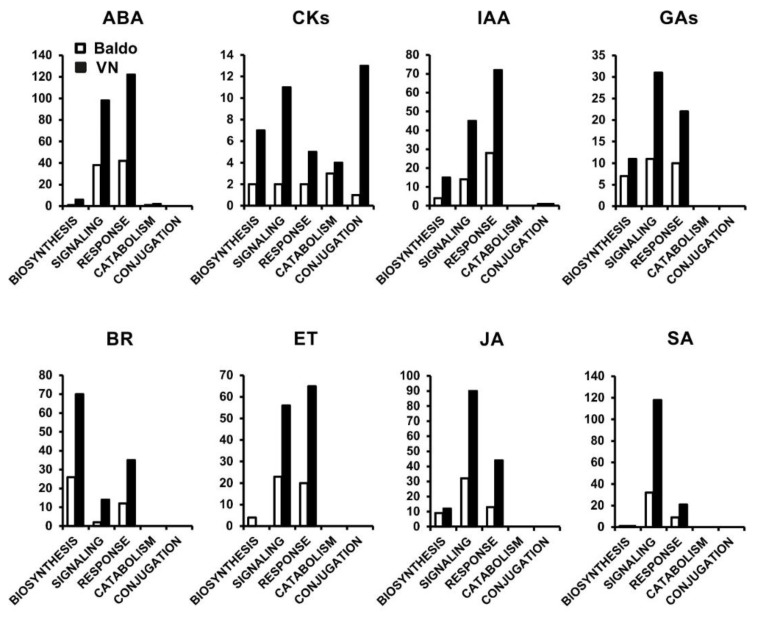
Number of DEGs involved in hormones metabolism in leaves of three-day treated plants. Genes belonging to gene ontology (GO) terms associated with hormonal biosynthesis, signaling, response, catabolism, and conjugation were gathered from RNAseq data [47].

**Figure 4:**
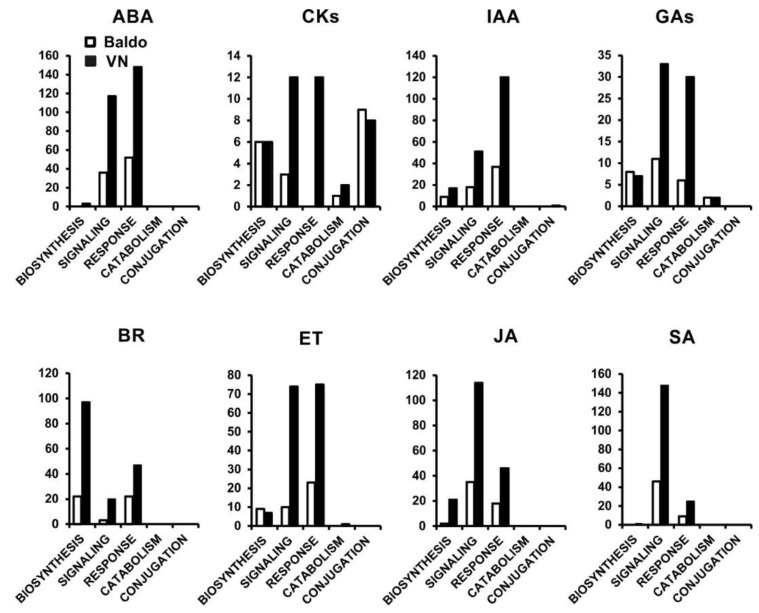
Number of DEGs involved in hormones metabolism in roots of three-day treated plants. Genes belonging to GO terms associated with hormonal biosynthesis, signaling, response, catabolism, and conjugation were gathered from RNAseq data [47].

We then gathered all the genes related to hormone metabolism in specific categories: biosynthesis, signaling, response, catabolism, and conjugation. Interestingly, almost all plant hormones were involved, even if to different extents. Figure 3 and Figure 4 show the number of regulated genes in leaves and roots, respectively, gathered for each hormone in the selected categories. The trend in the expression of genes reported in Figure 3 and Figure 4 is shown in Table S1, where the numbers of up- and down-regulated genes are reported.

A broader range of differentially expressed genes (DEGs) was present in the sensitive plants, suggesting once again a wider, not too specific, response to salt stress. Differences were indeed noticeable for some categories and in the proportions of up- and down-regulated genes.

As expected, functional categories involved in signaling and response to abscisic acid (ABA) were the most represented (Figure 3 and Figure 4) in both varieties. Genes of these categories coded mostly for protein phosphatases 2C, PYL receptors, and MYB-related transcription factors (Tables S3–S6, respectively). In leaves of both varieties, these genes were more up- than down-regulated (Table S1). In roots, the situation was slightly different. In fact, the tolerant variety showed the same leaf trend, which was inverted in the sensitive variety (Table S1). On the other hand, genes involved in the biosynthesis category were modulated only in VN plants, both in leaves and roots, suggesting a persisting ABA synthesis. Interestingly, one of these genes encoded for MHZ4 (LOC_Os01g03750), a homolog of Arabidopsis ABA4, reported to be involved in ABA biosynthesis mediated by ethylene [49] (Table S5).

Notably, few genes related to cytokinins (CKs) were found in both varieties. In the sensitive plants, the higher number of genes in the signaling and response categories suggested the presence of higher amounts of CK with respect to Baldo plants. However, in VN leaves, many genes involved in CK inactivation by conjugation (cytokinin-O-glucosyltransferases) were up-regulated, as plants were trying to lower the content of active CK (Table S5). Conversely, in salt tolerant plants, majority of genes involved in the synthesis (cytokinin riboside 5′-monophosphate phosphoribohydrolases) and sensing (type-A response regulator, MYB-type transcription factors) of CKs were down-regulated (Figure 3 and Figure 4, white bars; Tables S1, S3, and S4), whereas those involved in conjugation were up-regulated in roots (Figure 4; Tables S1 and S4), thus suggesting that tolerant plants were maintaining a low CK activity. As already observed, the trend in almost all the functional categories, emerging from the analysis of the sensitive variety, was not univocal; instead, a chaotic up and down-regulation was observed either in root or leaf (Figure 3 and Figure 4, black bars; Table S1).

Looking at the results of transcriptional changes in functional categories related to indole acetic acid (IAA), the biosynthesis, signaling, and response categories showed a similar trend of regulation in the two varieties with more genes modulated in sensitive plants (Figure 3 and Figure 4, black bars). At a closer look, a different proportion of up- and down-regulated genes was noticeable: more genes were down-regulated in VN roots compared to the same categories in the tolerant variety (Table S1). However, in roots of tolerant Baldo plants, a slight increase in synthesis and perception of IAA (auxin responsive factors, Table S4) was noticeable (Figure 3 and Figure 4, white bars; Table S1).

The analysis of genes related to gibberellins (GAs) demonstrated that the tolerant variety, at three days’ treatment, was beginning to recover growth after the arrest caused by salt stress. The GA biosynthesis and signaling categories were clearly up-regulated either in roots or leaves, whereas the response category was still down-regulated (Figure 3 and Figure 4; Table S1). Genes encoding gibberellin-20 oxidases and ent-kaurene synthases were up-regulated, whereas those coding for gibberellin receptors (GID1) were mainly down-regulated (Tables S3 and S4). In the sensitive variety, instead, non-univocal trend was present but an equal up- and down-regulation of genes in these categories was observed (Table S1).

By observing the trend in functional categories involving ethylene (ET), it appeared that this hormone was more present in the leaves of the sensitive variety when entering the senescence program after salt stress [47]. Genes involved in ethylene signaling, such as ethylene responsive transcription factors (ERFs), along with those involved in protein ubiquitination, were the most represented (Table S5)

In case of genes related to brassinosteroids (BR), a clear up-regulation of the biosynthesis category, represented by genes belonging to the cytochrome p450 family and genes encoding epimerases (Table S4), was present only in the roots of the tolerant variety (Table S1), even though VN plants showed a broader range of DEGs than Baldo plants (Figure 3).

Not particularly clear indications were recorded from genes related to salicylic acid (SA) and jasmonic acid (JA) even if transcriptional changes in some of the analyzed functional categories were present for both hormones. The number of DEGs in the signaling and response categories in VN plants (Figure 3 and Figure 4, black bars) suggested a conspicuous presence of SA and JA, perhaps due to the cell death program undergone by these plants. In fact, genes encoding for PCD/senescence-related genes were noticed (Tables S5 and S6), i.e., alpha-DOX2 (fatty acid alpha-oxidase dioxygenase 2 [50]) and BAX inhibitor 1 [51].

These results clearly depicted the defeat of VN plants that were unable to initiate a specific response, which eventually died by senescence. Despite the regulation of a huge number of stress-related genes (Figure 2) that indicated the sensing of the stress, the delay in the response, as previously reported [47], caused the accumulation of Na^+^ above toxicity thresholds and metabolism impairment in leaves. Many hormone-responsive genes appeared to be regulated in VN plants, suggesting a high accumulation of hormones like ABA, ET, and JA (Figure 3 and Figure 4; Table S1). These hormones are known to be involved in promoting leaf senescence. The role of ABA and ethylene in leaf senescence is well known [30], and ABA senescence-inducing capability has been also elucidated in rice [52]. Recently, Jasmonate was found to accumulate in stress-induced leaf senescence and to favor the process by the interplay with other hormones [53].

### 2.2. Changes in Hormonal Contents and Phenotypic Plasticity in Salt-Stressed Plants

Phenotypic plasticity is thought to be a useful resource for plants under stress. Shade avoidance and halotropism are some examples of the developmental reprogramming that plants can undergo in response to stress [12]. These changes are mediated by hormones in a complex interplay that scientists are only recently trying to understand.

In response to salt stress, we observed significant changes in the hormone-linked gene expression (Figure 2, Figure 3 and Figure 4). Phenotypic variations, along with differences in hormone contents, were thus estimated in our experimental system. Tolerant and sensitive plants were grown in hydroponics up to the vegetative stage V2 (fully expanded second leaf) [54]. Then, plants were treated with 100 mM NaCl, 10 mM Na_2_SO_4_, 20 mM MgCl_2_, and 10 mM CaCl_2_, for one and three days and the contents of ABA, IAA, CKs, and GAs were measured in leaves, stems, and roots.

In parallel, morphological parameters were evaluated in order to correlate hormone metabolism and plant plasticity. Three phenotypic responses, known to be affected during salt stress [5], were considered: (1) stomata aperture, and (2) shoot and (3) root growth.

Interestingly, significant changes in the levels of these hormones in salt-treated versus control plants were detected along with differences in morphological features when the two varieties were compared.

#### 2.2.1. ABA and CKs Levels Control Stomata Aperture

**Figure 5:**
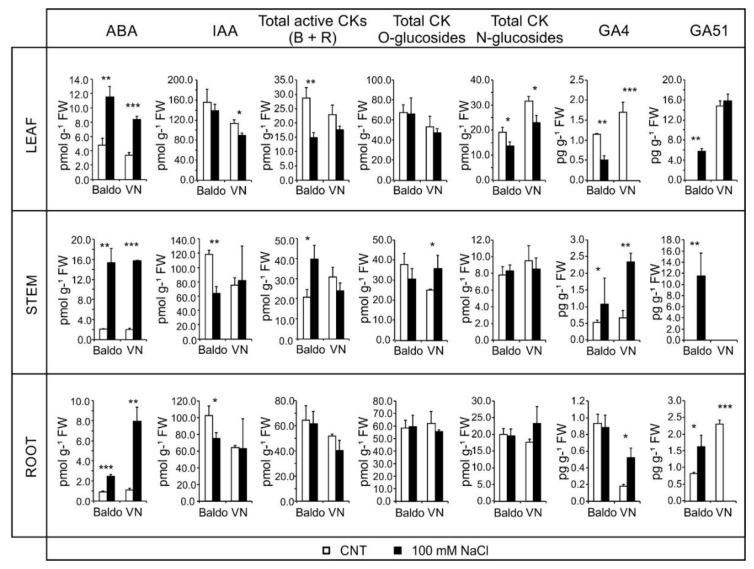
Hormone contents in plants treated for one day. Plants at the V2 stage of growth were treated with 100 mM NaCl. The content of main hormones in different organs was measured by mass spectrometry after one day. Data are expressed as mean ± SD, *n* = 6, * *p* < 0.05, ** *p* < 0.01, *** *p* < 0.001.

ABA content, as expected, increased rapidly in both varieties (3.71 times in Baldo and 4.86 times in VN after one-day salt treatment). It is interesting to note that about 50% of ABA total content was located in the stems of both varieties. The other 50% showed an equal partitioning between roots and leaves in VN (24.7% and 26.1%, respectively) and an uneven partitioning between the two organs in Baldo (8.5% and 39.3%, respectively) (Figure 5, Table S2).

**Figure 6:**
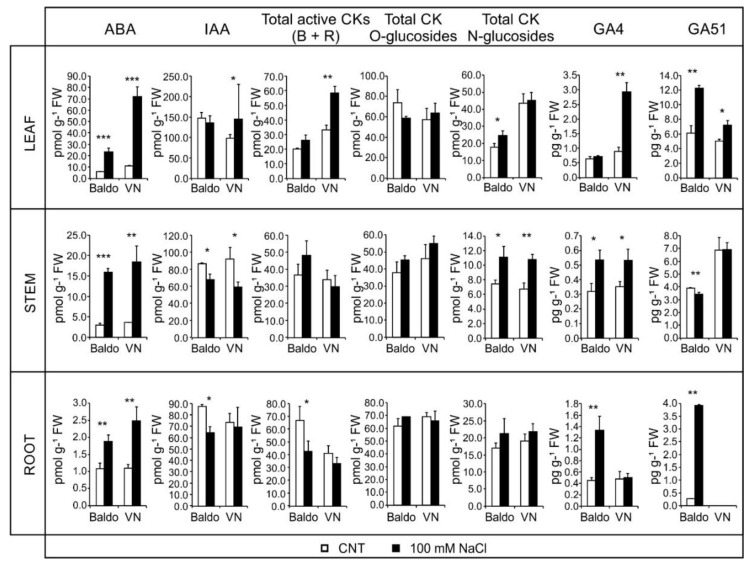
Hormone contents in plants treated for three days. Plants at the V2 stage of growth were treated with 100 mM NaCl. The content of main hormones in different organs was measured by mass spectrometry after three days. Data are expressed as mean ± SD, *n* = 6, * *p* < 0.05, ** *p* < 0.01, *** *p* < 0.001.

A further increase in ABA content was detected after three days of salt treatment, 4.04 times in Baldo and 5.88 in VN. At this second time point, higher levels of ABA were measured in leaves, reaching 77.5% of the total content in VN and 56.8% in Baldo (Figure 6, Table S2). Results showed that Baldo plants had a high level of ABA in leaves earlier than VN. Instead, at three days, VN leaves contained a huge amount of this hormone in comparison to Baldo.

ABA is involved in stomatal closure upon drought or salt stress. Recent findings demonstrated that CKs are involved in stomata opening, in contrast to the ABA action under drought stress [55]. To better understand the interplay between the two hormones, we measured the CK content and calculated the ratio [ABA]/[total active CKs], where total active CKs included all bases and ribosides forms of cytokinins. In leaves of tolerant plants, after one day of treatment, the total amount of active CKs decreased, and the ratio increased much more than in the sensitive plants (Figure 5).

At day three of treatment, the proportion was instead in favor of VN, even if active CKs increased, due to the huge accumulation of ABA in leaves. At this timepoint, in leaves of Baldo plants, a slight increase in N-glycosylated forms of CKs was observed, which could contribute to maintaining a low amount of total CKs.

Besides CKs, auxin was shown to be involved in the opening of stomata [39], but in our system, no differences were observed in its content between treated and untreated plants.

**Figure 7:**
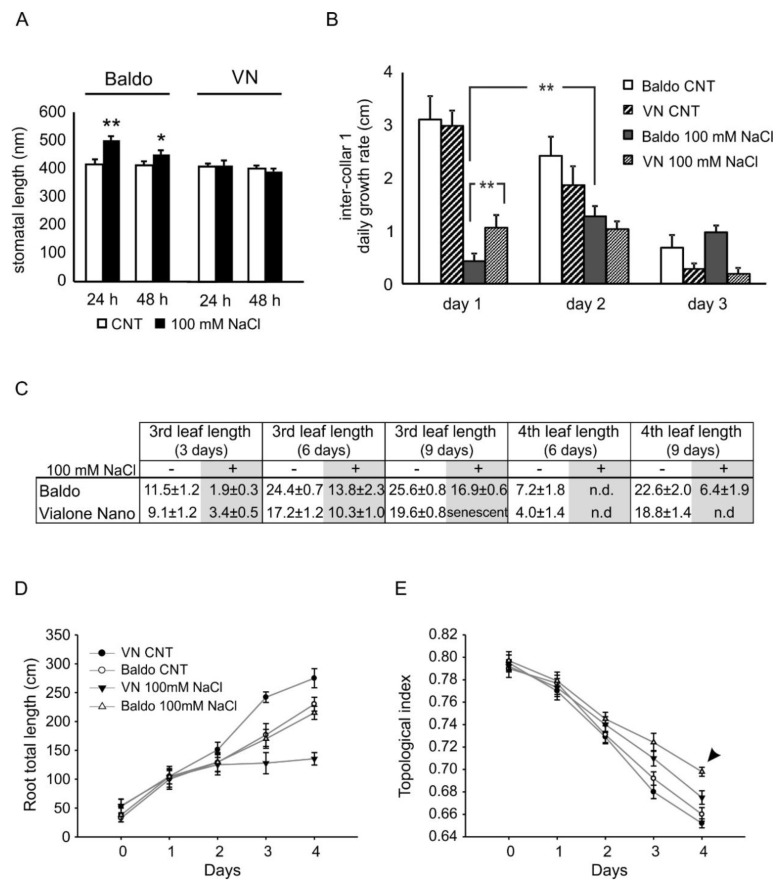
Stomata aperture and growth parameters measurements. (**A**) Stomata aperture was measured as the elongation of the stomatal complex. The longer the stomata, the smaller the stomata aperture. (**B**) Daily growth of the first inter-collar region. (**C**) Third and fourth leaf lengths at different timepoints under stress conditions. Data are expressed as mean ± SD, *n* = 6. * *p* < 0.05; ** *p* < 0.01. (**D**) Root total length and (**E**) topological index of the sensitive (black symbols) and tolerant (empty symbols) cultivars are shown in presence (triangles) and absence (circles) of salt. The tolerant cultivar shows a change in root structure architecture (RSA) in response to stress (E, arrow), while the sensitive plants display an irreversible growth arrest (**D**). Data are expressed as mean ± SEM, *n* = 9, *p* < 0.01.

By measuring the mean stomata length in the second leaf of Baldo plants one day after treatment, we observed a reduction in stomata aperture that was missing in VN plants. This condition was maintained on the second day of treatment [47] (Figure 7A). Unfortunately, in VN, most of the second leaf was yellowish after three days of treatment, making stomata aperture measurements unreliable at this timepoint.

The reduction in stomata aperture induced by ABA is a typical response of plants under water stress [25,26,27,28,29,30,31,32,33,34,35,36,37,38,39,40,41,42,43,44,45,46,47,48,49,50,51,52,53,54,55,56,57]. High soil salinity causes the decrease in the water potential at the root level and the loss of water (water stress). Limiting the transpiration by closing stomata helps in preserving the water content and metabolism. Moreover, the reduction in the transpiration stream can limit the Na^+^ uptake and translocation to leaves, thus reducing damage due to ion toxicity [58].

The capability of Baldo plants to maintain a high [ABA]/[total active CKs] ratio resulted in the reduction in stomata aperture, representing the possible mechanism by which Baldo plants preserved their relative water content and accumulated less Na^+^ in leaves [47].

Stomatal closure under drought conditions was regulated by ABA through the down-regulation of MYB60 genes in Arabidopsis and grape [59,60]. Consistently in Baldo leaves, two MYB60-like genes (LOC_Os11g03440 and LOC_Os12g03150) were strongly down-regulated in the group of ABA responsive genes (Table S3).

#### 2.2.2. IAA and CKs Levels Control Shoot Growth

In the shoots, differences were observed in IAA and CKs contents between the two varieties. Baldo showed a clear and consistent decreasing trend in the IAA content in the stem both at one and three days, whereas no significant differences were detected in leaves. In VN plants, IAA decreased in the stem only after three days of treatment. In leaves, the trend was not consistent: after an initial decrease, a slight increase was detectable after three days.

CK contents showed significant differences in Baldo plants at day one of treatment. Total active CKs increased significantly in the stem, whereas the level decreased in leaves, as previously reported. In VN plants, a slight increase in inactive O-glycosilated CKs was detected in the stem of one-day treated plants. After three days of treatment, a substantial increase in total active CKs was detected in VN leaves, whereas in Baldo leaves, N-glycosilated CK forms slightly increased.

Based on a detailed analysis of the growth parameters, we observed that the tolerant variety Baldo showed a rapid reduction in growth rate (13.8% of the control; Figure 7B, day one) at the level of the first inter-collar (inter-collar 1; Figure S1). By contrast, in the sensitive variety VN, the reduction in the first inter-collar growth rate was slower (35.5% of the control, Figure 7B, day one), Stem growth recovered in the next days (Figure 6, days two and three), and new leaves eventually emerged in salt-tolerant plants (Figure 7C), whereas leaf yellowing ensued, and no new leaves appeared in sensitive plants (Figure 7C, senescent). Interestingly, among genes involved in PCD/senescence, we found three of them previously shown as regulated by JA: LOC_Os12g26290, encoding the fatty acid alpha-oxidase dioxygenase 2 (alpha-DOX2 [50]); LOC_Os02g03280, coding for BAX inhibitor 1 [51]; and LOC_Os11g45740, which encodes a MYB transcription factor [61]. This finding implicated the intervention of JA in salt-induced senescence.

The cytokinin-auxin interaction is thought to be involved in shoot meristem maintenance [35,62]. High levels of CK are responsible for stem cell maintenance in the shoot meristems and auxin was shown to counteract this function by inhibiting CK synthesis in maize [35,63]. As observed above for stomatal closure, the key factor in determining the fate of shoot growth is not the absolute content of each hormone, but the ratio between them. The ratio between the two hormones in the stem of tolerant plants was indeed largely in favor of CKs, thus suggesting a role in the maintenance of meristems and possibly in the fast-recovering of growth observed after the initial growth restriction. Moreover, with auxin also being involved in cell division, its reduction can be responsible for the growth repression observed at one day in salt-tolerant plants.

Notably, treated Baldo plants resumed growth but to a much lesser extent in the stem, where the second inter-collar region had a growth rate of about 0.15 cm/day (3 cm/day for control plants). Leaves instead grew at a similar rate as those of untreated plants, although with a delay and with shorter length (Figure 7C). Interestingly, the hormonal balance in leaves of three-day treated Baldo plants was similar to that of untreated plants.

Regarding active CKs, a clear difference was noted in the proportion of the single isoforms in the two varieties. In the stem of Baldo treated plants, the majority of active CKs, increased under stress, were represented by the riboside forms (Table S2). It is well known that xylem sap levels of CKs (in particular tZR) are reduced in response to different abiotic stresses, drought *in primis*. On the other hand, the increase in endogenous CKs, by overexpressing isopentenyl transferase (IPT) encoding genes led to contrasting results, from improved drought tolerance to increased sensitivity (reviewed in [64]). We can hypothesize that high levels of cytokinin ribosides coming from the root through the xylem represent a fast-deliverable storage for CKs.

#### 2.2.3. ABA, IAA and CKs Crosstalk in Shaping the Root Morphology

In the roots of tolerant plants, significant variations were observed in ABA and IAA levels at one day of treatment, and in total active CKs forms at three days. After one day of treatment, in Baldo roots, ABA was well lower than in VN, and IAA decreased. After three days, ABA levels were comparable in the two varieties, but in Baldo roots, IAA and active CKs decreased.

Total root length and topological index were determined using WinRhizo software. Morphological analyses showed that tolerant and sensitive varieties displayed contrasting root patterns in response to salt treatment (Figure 7D,E). In the sensitive variety, a significant decrease in total root length was observed two days after the salt treatment compared with that in the control group, leading to a growth arrest in the following days. By contrast, in the tolerant variety, no differences in total root length were observed after salt treatment (Figure 7D).

Root architecture was also analysed to identify specific root characteristics of the tolerant variety. The topological index did not differ significantly between Baldo and VN control roots (Figure 7E), with both showing a dichotomous pattern. However, significant changes (*p* < 0.05) in the root topology of the tolerant variety were observed four days after salt treatment. As VN roots stopped growing and maintained their initial structure (topological index: 0.67 ± 0.006), the roots of Baldo plants expressed a more herringbone topological pattern (topological index: 0.70 ± 0.004; Figure 6D, arrow tip).

Li et al. [65] reported that ABA can either promote or inhibit root growth under water stress in Arabidopsis, depending on its concentration. The authors demonstrated that low amounts of ABA led the roots to grow, whereas high ABA concentrations caused a quiescence. The latter phenomenon was previously reported by Rowe et al. [18] and was shown to be related to the interplay of ABA with auxin and ethylene [18,43]. At low ABA concentrations, instead, the stimulation of the growth was not related to ethylene signaling, but possibly to auxin transport regulation by ABA [18,35]. Auxin was involved in the formation of lateral root primordia (LRP), and endodermal ABA was proven to counteract LRP establishment under water stress by hampering auxin polar transport [12,31,43,66].

In Baldo salt tolerant plants, under salinity conditions, roots continued to grow at the same rate of the control plants, but the structure shifted toward a more herringbone pattern: the main roots grew, and no lateral roots were initiated. The ABA and IAA levels in Baldo roots were much lower than in the sensitive plants and ethylene signaling and response categories of genes were much less represented, indicating a possibly lower content of ethylene. These findings support the hypothesis that the shift toward a more herringbone structure of roots of tolerant plants was related to the inhibition of lateral root formation and to the increase in main root cell elongation.

#### 2.2.4. Role of GAs in Growth Regulation in Response to Salinity: From Quiescence to Recovery

Recent findings set gibberellins at the crossroad of hormone interaction in growth responses related to stresses [19,43]. By measuring the GAs levels in stems, roots, and leaves of the two varieties, GA4 was confirmed as the active form present in rice (Table S2) [67,68]. The total amount of this active form did not change after one day’s treatment, but the hormone localization was different (Figure 5). A reduction in the GA4 level was observed in the leaves of both varieties, along with an increase in the stem (2 times in Baldo, 3.4 times in VN). An increase in GA4 levels was present only in VN root.

Clearer differences between the two varieties emerged after three days’ treatment when the GA4 total level almost doubled (1.83 times in Baldo, 2.30 times in VN). In the tolerant variety, the increase was essentially in the roots (3.4 times); in the sensitive one, it occurred in the leaves (3.2 times) (Figure 6).

The levels of inactive GA51, obtained by 2beta-hydroxylation of GA9, increased rapidly and consistently after one-day treatment, particularly in the leaves, roots, and stems of Baldo (Figure 5), in which shoot growth was rapidly arrested (Figure 7B), which occurred only slightly in VN leaves. GA51 maintained its levels above control after three days’ treatment, thus playing an important role in reducing the active GA4 in the plant under salt stress (Figure 6 and Table S2).

Salt stress causes an initial phase of quiescence during which meristem activity is reduced. DELLA protein stabilization or GA degradation enzymes activation by ABA [19,43] and IAA reduction are involved in this phase. After the quiescence period, plants with less damage resumed growth. In our system, tolerant plants seemed to repress the growth by increasing GA51 levels and reducing IAA accumulation. The concomitant presence of active CKs and GAs could, instead, help to preserve meristem activity and resume growth (Figure 7) [18,35,43].

In roots, the increase in GA51 and the decrease in IAA and CKs could be involved in maintaining the quiescence of lateral root primordia meristems in tolerant plants. The up-regulation of brassinosteroids biosynthesis genes (Table S1) and the increase in GA4 content (Figure 6) in three-days treated Baldo plants could be involved in the recovering of cell cycle activity. Roots of tolerant plants then continued to grow but changed topology (Figure 7E). In sensitive plants, instead, the presence of active GA4 and the lack of GA51, along with high ABA and ethylene levels, could be responsible for the initial growth and lateral roots formation followed by a complete inhibition of cell division and/or elongation in the next days (Figure 7E).

## 3. Materials and Methods

### 3.1. Plant Material

Dehulled seeds of Vialone Nano (VN) and Baldo Italian rice varieties (*O. sativa* L. spp. japonica “temperate”) were sterilized for 1 min in 70% ethanol and rinsed 5 times with deionized water. Seeds were sown on water-wetted filter paper in Petri dishes and left to germinate for 48 h at 24 °C in the dark. Uniformly germinated seedlings were transferred to agar-filled (0.55% *w*/*v*) seed holders in an Araponics (hydroponic growing) system (www.araponics.com) with a modified Hoagland solution [47,69]. Before treatment, plants were grown in stationary conditions, until the vegetative stage V2 (collar formation on the second leaf) [54] at approximately 6 days in a growth chamber at 26/21 °C, with a 16/8 h photoperiod, an approximate relative humidity (RH) of 70%, and light of 200 µmol photons m^−2^·s^−1^. A saline solution similar to diluted sea water was added to the hydroponics at the final concentration of 100 mM NaCl, 10 mM Na_2_SO_4_, 20 mM MgCl_2_, and 10 mM CaCl_2_. Plants were then grown for 1 to 6 days.

### 3.2. Transcriptomic Analysis

For RNA-seq analysis, 3-day treated samples (leaves and roots) were frozen in liquid nitrogen. To minimize the effect of individual polymorphisms, each biological replicate was a mix of 6 plants. Three biological replicates were used in each experiment. Samples were sequenced on a HiSeq2500 platform (IGATech, Udine, Italy) and data were processed with a home-made pipeline described in Formentin et al. [47]. Briefly, raw reads were mapped by TopHat [70] on the *Oryza sativa* v. Nipponbare genome, downloaded from the MSU Rice Genome Annotation Project (version 7.0) [71]. Read counts were computed using bedtools [72]. The entire set of transcripts was annotated with the Argot web server [73,74,75] (http://www.medcomp.medicina.unipd.it/Argot2/), which assigned Gene Ontology terms to each input sequence (Gene Ontology Annotation database downloaded on 2013-12-29, PFAM release 27.0). This procedure provided novel annotations for many genes with unknown function. Differential expression analysis was carried out using edgeR Bioconductor package v. 3.8.6 [76]. Finally, we computed a likelihood ratio test and considered differentially expressed those genes having a *p*-value greater than 0.05 after a Benjamini and Hochberg correction for multiple testing [77].

The datasets used for this study can be found in the Gene Expression Omnibus (GEO) under accession number GSE109341 [47].

### 3.3. Root Morphology Analysis

Root morphological traits were evaluated with a scanner-based image analysis system (WINRHIZO Pro; Regent Instruments, Quebec, Canada) that scanned, digitized, and analysed root samples. The topological index (TI) was calculated as the ratio of the natural logarithm of the altitude (the most links between an external link and the base link) and the natural logarithm of the magnitude (terminal root sections between the meristem and the nearest branching point). Values of TI close to 0 indicate a dichotomous branching pattern, whereas those close to 1 reveal a herringbone pattern [78,79].

### 3.4. Hormones Analysis

Six-day-old seedlings were grown with or without 100 mM NaCl, 10 mM CaCl_2_, 20 mM MgSO_4_, and 10 mM Na_2_SO_4_. Leaves (blade and sheath), stems, and roots (washed carefully) were collected after 1 and 3-day salt stress for analysing hormonal content. IAA, ABA, and CKs were analysed as in Flokovà et al. [80]. GAs were measured according to Urbanovà et al. [81].

### 3.5. Stomatal Aperture Measurements

The analysis was performed on the second leaf of plants at stage V2, treated or untreated for 24 h and 48 h. About 20 stomata/leaves were imaged (Leica 5000b, Leica Microsystems, Wetzlar, Germany, 100X objective). The stomatal length was measured by the mean of ImageJ2 [82].

### 3.6. Statistical Analyses

Student’s *t*-tests were applied to experiments with a sample number greater than 30. Wilcoxon-Mann-Whitney tests were applied for *n* < 30.

## 4. Conclusions

In this work, we report data supporting the hypothesis that a fast and strict control over relative hormone amounts was pivotal in determining salt tolerance in rice. Recently, fast molecular and hormonal regulation in response to salt stress was associated to tolerance in the halophyte *Thellungiella salsuginea* [83]. The analysis of growth parameters in two rice varieties differing in salt sensitivity unveiled that the first response of both sensitive and tolerant plants was a growth restriction of the shoot, determined by the increased ABA concentration in the stem. In tolerant plants, the growth reduction in the first inter-collar region was more pronounced and transitory. A slight recovery was observed in the next days, and new leaves emerged from the stem. The extent of the growth reduction was probably related to the decrease in the stem cell division rate due to the decline of IAA and active GAs (low GA4/GA51), and the consequent meristem reduction [35,43]. The recovery phase, instead, could be related to both high levels of active CKs, known to maintain meristem activity [35], and the increase in the ratio between GA4 and GA51. The latter triggered the division of stem cells. However, the plant recovery was not complete, neither at the shoot nor at the leaf level, as shown by the length of the leaves after nine days of stress (Figure 7C) or the shortness of the second inter-collar region (Figure 1D). The loss of turgor, experienced in the initial phase of stress response (quiescence phase) along with the reduction in the number of cells in meristems, could perhaps be responsible for the observed limited growth recovery.

Conversely, sensitive plants, despite initially entering the quiescence phase, did not recover at all, and underwent leaf senescence and death. The ABA content in VN leaves was much higher, and ethylene responsive genes were much more represented than in Baldo plants. This is in line with the role of the two hormones in plant senescence [84]. Moreover, CKs and GAs levels were very high. We do not know if this is an attempt to counteract the senescence process [38], or the result of the impaired metabolism and uncontrolled gene expression.

One of the main findings of this work was the uninterrupted root growth in tolerant plants and the remodeling of the root system architecture (RSA). Sensitive plants, as expected, demonstrated slowed root growth, after two days, and eventually stopped when leaf senescence arose, after day three. Roots of the tolerant variety, in which both ABA and IAA were less concentrated with respect to VN plants, continued to grow at the same rate of the roots of the control plants, with the only difference being that lateral root formation was inhibited. This is partially in contrast to what has been reported for Arabidopsis [31], where both lateral and primary roots were affected by salt, even if to different extents (86% and 46% growth reduction, respectively). Moreover, in rice, the reduction in root length was observed under high salinity [85]. The elongation of the seminal root was instead observed under drought stress in rice [86]. We cannot exclude the fact that the roots of Baldo plants elongated in response to an osmotic stress, exploring deeper soil layers in search for water. Another possible explanation is that roots are avoiding the salt (hydrotropism). Unfortunately, only little is known about RSA remodeling of crops during salt stress and further research is needed. In our system, however, the root level of ABA and IAA seemed to have a role in shaping RSA. As in salt-sensitive plants, the only of effect of the high content of ABA was stopping the root elongation.

Taken together, our results indicate that salt tolerance in rice is promoted by the fast synthesis and partitioning of hormones in different organs, and highlights the importance of the fine tuning of hormonal ratios at the tissue and cell level. In particular, changes in GAs levels are of some significance and deserve future investigation. In addition, further studies will address the molecular basis of the interplay among ABA, CKs, and IAA in inducing salt tolerance by exploring common gene regulation networks at a cell-specific level.

## Supplementary Materials

The following are available online at http://www.mdpi.com/2223-7747/7/3/75/s1, Figure S1: Representation of a rice seedling, Table S1: Number of up- and down-regulated genes in each category shown in Figure 3 and Figure 4. Differences are highlighted in grey; Table S2: ABA, IAA, CKs, and GAs content in leaves, stems, and roots, Table S3: List of genes from Baldo leaves shown in Figure 3 and Table S1, Table S4: List of genes from Baldo roots shown in Figure 4 and Table S1, Table S5: List of genes from VN leaves shown in Figure 3 and Table S1, Table S6: List of genes from VN roots shown in Figure 4 and Table S1.
