# Supplementary figures and images for: Fast Regulation of Hormone Metabolism Contributes to Salt Tolerance in Rice (Oryza sativa spp. Japonica, L.) by Inducing Specific Morpho-Physiological Responses
Source: Plants (Basel). 2018 Sep 15;7(3):75. doi: 10.3390/plants7030075 (PMC6161274; doi:10.3390/plants7030075)

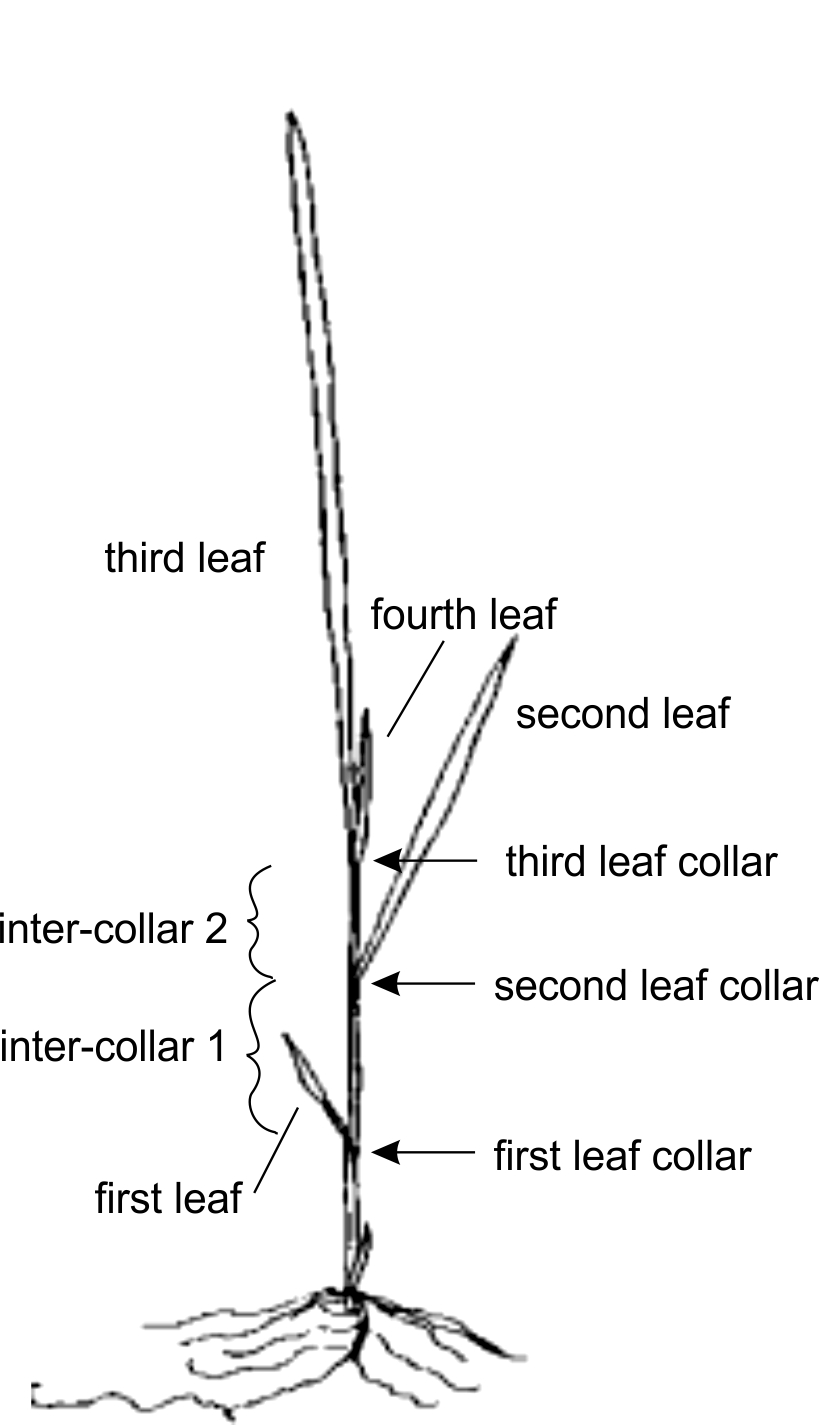

Supplement: Supplementary file 1 [file plants-07-00075-s001.zip › Figure S1.jpg]
